# Recent advances in rhythm control for atrial fibrillation

**DOI:** 10.12688/f1000research.11061.1

**Published:** 2017-10-03

**Authors:** Richard Bond, Brian Olshansky, Paulus Kirchhof

**Affiliations:** 1 University Hospitals Birmingham NHS Foundation Trust, Birmingham, UK; 2 University of Iowa, Iowa City, USA; 3 Department of Cardiovascular Medicine, Hospital of the University of Münster, Münster, Germany; 4 Atrial Fibrillation NETwork (AFNET), Münster, Germany; 5 Institute of Cardiovascular Sciences, University of Birmingham, Birmingham, UK; 6 Sandwell and West Birmingham NHS Trust, Birmingham, UK

**Keywords:** Atrial Fibrillation, Sinus rhythmcontrol, ablation

## Abstract

Atrial fibrillation (AF) remains a difficult management problem. The restoration and maintenance of sinus rhythm—rhythm control therapy—can markedly improve symptoms and haemodynamics for patients who have paroxysmal or persistent AF, but some patients fare well with rate control alone. Sinus rhythm can be achieved with anti-arrhythmic drugs or electrical cardioversion, but the maintenance of sinus rhythm without recurrence is more challenging. Catheter ablation of the AF triggers is more effective than anti-arrhythmic drugs at maintaining sinus rhythm. Whilst pulmonary vein isolation is an effective strategy, other ablation targets are being evaluated to improve sinus rhythm maintenance, especially in patients with chronic forms of AF. Previously extensive ablation strategies have been used for patients with persistent AF, but a recent trial has shown that pulmonary vein isolation without additional ablation lesions is associated with outcomes similar to those of more extensive ablation. This has led to an increase in catheter-based technology to achieve durable pulmonary vein isolation. Furthermore, a combination of anti-arrhythmic drugs and catheter ablation seems useful to improve the effectiveness of rhythm control therapy. Two large ongoing trials evaluate whether a modern rhythm control therapy can improve prognosis in patients with AF.

## Introduction

Atrial fibrillation (AF) remains a difficult management problem. The restoration and maintenance of sinus rhythm—rhythm control therapy—can markedly improve symptoms and haemodynamics for patients who have paroxysmal or persistent AF, but some patients fare well with rate control alone ^1– 4^. AF can be a cause for impaired exercise capacity ^3^, dementia, hypotension, syncope, dyspnoea, and heart failure, among other problems ^1, 5^, and is a major risk factor for ischaemic stroke ^1– 4^. In addition, AF is associated with a higher risk of death in long-term follow-up ^2, 4– 6^.

AF management involves reducing stroke risk and improving symptoms and functionality. Oral anticoagulation (OAC) can prevent the majority of ischaemic strokes from AF in at-risk patients ^2, 7^. Controlling the ventricular rate during AF can improve patients’ symptoms, but the maintenance of sinus rhythm is necessary for complete improvement in some patients. Techniques to restore sinus rhythm include pharmacological cardioversion with anti-arrhythmic drugs (AADs) and electrical cardioversion (performed with external defibrillation pads or an internal cardioversion catheter) ^8^. Catheter and surgical ablation can prevent AF recurrence ^2, 9^. This review will discuss the current and recent advances in catheter ablation for rhythm control of AF.

## Classification of atrial fibrillation

AF is classified as first detected episode, paroxysmal (spontaneously terminates or cardioverted in less than 7 days), persistent (AF lasting for more than 7 days or requiring pharmacological or electrical cardioversion), longstanding persistent (continuous AF for over 1 year when it is decided to adopt a rhythm control strategy), or permanent (when AF is accepted and rhythm control is no longer pursued) ^2, 10^. These definitions are used to guide treatment options for patients and also inclusion criteria for clinical trials ^11, 12^.

AF is often a chronic progressive disease, and animal studies have shown that “AF begets AF” ^13, 14^. In many patients, AF progresses as per currently accepted definitions; however, studies using implantable devices have shown that in some patients AF remains paroxysmal rather than progressing and persistent AF may regress to paroxysmal ^15, 16^. Furthermore, data from continued atrial rhythm monitoring using implantable devices have shown that a patient’s clinical AF classification poorly reflects the temporal persistence and suggests that the AF burden does not differ between patients with paroxysmal or persistent AF ^17^. This may have implications for ablation strategy and inclusion criteria in clinical trials.

## Indications for catheter ablation of atrial fibrillation

Catheter ablation can be useful in patients with symptomatic paroxysmal, persistent, and longstanding persistent AF that is refractory to or where there is an intolerance to AADs ^2, 9^. Catheter ablation is more effective than AADs in maintaining sinus rhythm ^18– 22^. Catheter ablation is also effective for restoring sinus rhythm in patients with AF and heart failure, improving left ventricular ejection fraction and quality of life ^23, 24^. There is no current indication for catheter ablation to prevent cardiovascular outcomes or desired withdrawal of anticoagulation ^2^. Patients are anticoagulated for the procedure either with uninterrupted warfarin aiming for an international normalised ratio of 2–3 or with an interrupted novel OAC (NOAC). Recent published trials comparing uninterrupted warfarin with uninterrupted NOACs for catheter ablation have had similar safety profiles ^25, 26^, and another trial comparing uninterrupted apixaban with uninterrupted warfarin is ongoing ^27^. Anticoagulation is continued for at least 8 weeks after the procedure. The decision to stop anticoagulation is based on the risk profile of the patients using general anticoagulation guidelines (for example, CHADS _2_-VASc2) rather than the presumed rhythm outcome ^2^. However, ongoing trials are investigating stopping anticoagulation after a successful ablation ^28^.

## Catheter ablation of atrial fibrillation

AF is often initiated by ectopic beats arising from the pulmonary veins (PVs), although non-PV triggers (such as the superior vena cava [SVC], posterior left atrial free wall, crista terminalis, coronary sinus, and ligament of Marshall) have been described by several studies ^29– 34^. Furthermore, other arrhythmia mechanisms, most notably micro-re-entry and other re-entrant patterns of atrial excitation, contribute to the initiation and maintenance of AF ^35^. The first successful reports of catheter ablation of AF targeted the triggering ectopic beats originating from the PVs preventing AF ^29, 30^. However, ablation within the PVs was associated with PV stenosis. It was recognised additionally that AF could also be initiated and maintained by PV antral tissue ^36– 38^. This led to electrical isolation of the PVs from the left atrium (PV isolation [PVI]) by either segmental PV ablation or, more commonly, wide area circumferential ablation (WACA) isolating the right and left PVs in pairs some distance from the venous tissue guided by 3D electro-anatomical mapping (EAM) ^39, 40^. 3D EAM uses an electromagnetic or impedance-based catheter location method to create a 3D anatomic shell of the atria ^41^. 3D EAM allows accurate visualisation of catheters within the anatomical shell without the use of fluoroscopy, which has led to a reduction in fluoroscopy time, procedure time, and radiation dose to the patient and operator ^42– 45^. There are three main mapping systems available: CARTO ^®^ (Biosense Webster, Diamond Bar, CA, USA), EnSite™ NavX™ (St. Jude Medical, Sylmar, CA, USA), and Rhythmia™ (Boston Scientific, Marlborough, MA, USA).

PVI remains the cornerstone of the AF ablation technique and is most commonly performed by point-by-point radiofrequency ablation ^46, 47^. However, whilst acute PVI may appear to be present after lesion delivery, there may be PV connection that is not evident because of local oedema masking conduction rather than actual tissue necrosis during the ablation ^48^. Furthermore, suboptimal contact force between catheter tip and atrial tissue during ablation is believed to reduce efficacy, although formal testing of this hypothesis is awaited. Contact force technology has been developed to allow instant feedback to the operator to allow adequate contact to deliver durable lesion without excessive force, which could lead to mechanical injury. Contact force technology has led to a significant reduction in AF recurrences and has reduced fluoroscopy and procedure times ^49^. Point-by-point radiofrequency ablation is complex and technically demanding, requires extensive training, and is largely limited to specialist centres ^47, 48^. The most commonly used alternative to point-by-point radiofrequency ablation is the cryoballoon ablation catheter, which creates a circular lesion (producing necrosis by freezing) around each PV and is associated with similar outcomes in paroxysmal AF ^47, 50^. Cryoballoon ablation is a simpler technique that can be associated with shorter procedural times but increased fluoroscopy times ^47, 51^. Cryoballoon ablation is now commonly used to isolate the PVs. Other balloon-mounted technologies developed for AF ablation are the high-intensity focussed ultrasound (HIFU) balloon, radiofrequency “hot” balloon, and visually guided laser balloon (VGLB) ^48, 52, 53^. Transvenous HIFU was demonstrated to be effective but was removed from the market owing to an unacceptable complication rate, including fatal atrial-oesophageal fistulas ^9^, but there may be a role for epicardial HIFU in surgical AF ablations ^54^. The radiofrequency “hot” balloon has shown promise but is still undergoing clinical trials. VGLB has been approved for use in Europe and permits direct visualisation of the target atrial tissue during ablation and is associated with good short-term outcomes ^48, 55^.

## Techniques for atrial fibrillation ablation

Durable PVI is accepted as an effective treatment for paroxysmal AF with 60–80% maintenance of sinus rhythm at 1 year and approximately equal to 50% at 5 years after a single procedure ^47, 50, 56– 58^. Ablation of non-PV triggers (SVC, posterior left atrial free wall, crista terminalis, coronary sinus, and ligament of Marshall) is usually performed if there is recurrent AF despite isolated PVs. Non-PV triggers can be identified by using isoproterenol infusion, isoproterenol and adenosine infusion, or rapid atrial pacing ^31, 32, 59, 60^. Ablation of non-PV triggers that still induced AF, after PVI, improved ablation outcomes in normal hearts and those with reduced ejection fraction ^59, 60^.

Results in those patients presenting with non-paroxysmal AF have lower success rates: 36–60% maintenance of sinus rhythm at 1 year and 20–42% long term after a single procedure ^12, 57, 61– 63^. Of note, some patients presenting with persistent AF have good long-term outcomes after AF ablation whereas other patients who present with paroxysmal AF have frequent and early recurrences. The structural remodelling that takes place in non-paroxysmal AF creates a substrate that maintains the arrhythmia and is believed to underlie recurrences when isolation of the PVs was successful ^64^. Previous ablation techniques have aimed to target this substrate (substrate modification) in addition to PVI by either ablating complex atrial signals, so-called complex fractionated atrial electrograms (CFAEs), or creating linear lesions in the left atrium—a roof line connecting the lesions around the left and right upper superior veins, a mitral isthmus line connecting the mitral valve annulus and left inferior PV, and an anterior line connecting either the anterior left upper PV or the right upper PV to the mitral valve annulus ^9, 12^. In longstanding persistent AF, a “stepwise” approach has been described ^65^. This starts with PVI followed by isolation of other thoracic veins—superior vena cava and coronary sinus. This is followed by ablation of CFAEs and then lines including a cavotricuspid line, roof line, and mitral isthmus line. At each step, if there is cardioversion to sinus rhythm, then ablation is stopped. Ablation techniques for persistent AF have longer procedure times and are associated with left atrial macro re-entrant tachycardia in 25% of patients ^2^.

Recently, the Substrate and Trigger Ablation for Reduction of Atrial Fibrillation Trial Part II (STAR AF 2 trial) compared three approaches to the ablation of persistent AF: PVI alone, PVI plus CFAEs, and PVI plus a roof and mitral isthmus line ^12^. The results of this study were quite surprising and showed no difference in outcomes between the three ablation strategies, illustrating the effectiveness of PVI for sinus rhythm maintenance in patients with AF. After 18 months of follow-up, 59% of patients assigned to PVI alone were AF-free compared with 49% of patients assigned to PVI plus CFAE ablation and 46% of patients assigned to PVI plus a roof and mitral isthmus line. A meta-analysis was performed incorporating data from STAR AF 2 as well as nine other studies with 1,821 patients included ^66^. In comparison with PVI alone, CFAE ablation and linear lesions offered no significant improvement in arrhythmia-free survival. This landmark trial has changed the way many perform ablations for persistent AF, supporting the use of PVI alone as a first-line therapy, which in the future could be performed by cryoballoon or VGLB ^67^. In fact, in a recent survey, the majority of centres (67%) were performing PVI alone as a first procedure in persistent AF, and over half of centres surveyed responded that the STAR AF 2 results had changed their strategy for persistent AF ablation ^68^. New ablation strategies for the ablation of persistent AF continue to be developed. A technique to target sources or “rotors” of AF using a multi-electrode basket catheter showed initial promise with at least 80% freedom from AF at a year ^69, 70^; however, these results have not been replicated ^71– 74^. Interestingly, the CONFIRM (Conventional Ablation for Atrial Fibrillation With or Without Focal Impulse and Rotor Modulation) study also demonstrated that 45% of AF sources were coincidentally ablated with conventional ablation for persistent AF (WACA and roof line), which may also explain why some patients do well after PVI whereas others may need more extensive ablation ^70^. A non-invasive vest comprising 252 body surface electrodes has also been developed to identify rotors; this technique records atrial epicardial electrograms, and a non-contrast computed tomography (CT) scan is used to produce anatomy and electrode position ^75, 76^. Another strategy recently described is scar homogenisation, where low-voltage areas in the atria thought to represent fibrosis are ablated ^77, 78^. These ablation strategies are still under development and have not been compared with PVI alone in randomised controlled multicentre trials. At present, PVI as first-line therapy for persistent AF is recommended ^2^.

## Complications of catheter ablation of atrial fibrillation

A total of 5 to 7% of patients will suffer a complication after catheter ablation of AF ^2, 79^. The most frequent complications are related to vascular access and usually can be managed conservatively ^80^. The most important severe complications are stroke/transient ischaemic attack (<1%), cardiac tamponade (1%), and phrenic nerve injury (0.001–3% depending on ablation energy used). PV stenosis, atrio-oesophageal fistula, and death are all rare ^2, 12, 47, 79, 81^. Vascular access complications potentially can be reduced by using ultrasound to image the femoral veins ^80^. Atrio-oesophageal fistulas are difficult to diagnose. Patients present with infection without a clear focus, pleuritic chest pain, stroke, or convulsions ^82^. Transoesophageal echo should be avoided, and diagnosis is by CT scan with emergent cardiac surgery as the treatment of choice, although temporary stenting of the oesophagus has been used in early diagnoses ^83, 84^.

## Surgical ablation of atrial fibrillation

The Cox maze procedure for the surgical treatment of AF was first used in 1987 and involves the creation of atrial incisions (around the PVs and posterior left atrium, connecting to the mitral annulus, cavotricuspid isthmus, and cavocaval connection, and exclusion of the left atrial appendage), which prevents atrial re-entry and allows the sinus impulse to activate the entire atrial myocardium ^85^. The long-term outcomes of 198 patients who underwent surgery, either as a standalone treatment for AF or in conjunction with other cardiac surgery, are excellent ^86^, although the rhythm follow-up has been less rigorous than in controlled clinical trials. Despite its efficacy, the original Cox maze procedure is complex and technically difficult and is associated with a high incidence of major complications (12%), including three perioperative deaths and three perioperative strokes ^9, 86^. This has led to less-invasive techniques and hybrid procedures. Atrial incisions have been replaced with linear lines of ablation with radiofrequency energy, cryoablation, or HIFU and can be performed via mini-thoractomy ^2, 9^. In one trial, the results of minimally invasive surgical ablation were better than catheter ablation but with a significant increase in complications ^87^. In an attempt to improve outcomes further, a “hybrid” approach, which combines a thorascopic epicardial with percutaneous endocardial catheter ablation, has recently been described ^88, 89^. Initial results have been excellent, but more trials are needed and the complication rates from surgery may make the procedure prohibitive for standalone AF but may be useful in those undergoing other cardiac surgery.

## Hybrid rhythm control therapy

AADs are continued after a catheter ablation for 8–12 weeks during the so-called “blanking period” ^90^. This strategy is used to reduce the early recurrence of atrial tachyarrhythmias whilst scar formation from ablation lesions takes place ^2, 91^. Evidence supporting this strategy is provided by the AMIO-CAT (amiodarone after catheter ablation for atrial fibrillation) study, which randomly assigned patients to either short-term amiodarone or placebo and demonstrated a significant reduction in early recurrence of AF during the blanking period in the amiodarone group ^91^. After the blanking period, it is common practice to stop AADs. However, catheter ablation with continued use of an AAD is associated with fewer recurrences of atrial tachyarrhythmias, and this includes AADs previously reported as ineffective ^90, 92^. Therefore, it would seem reasonable to continue on AADs after an ablation or to restart AADs if there is a single recurrence of AF, although in reality most patients would like to stop their AADs.

## Future directions

Pursuing sinus rhythm in patients with AF is recommended for those who are symptomatic, although we still do not know whether it confers any prognostic benefit (that is, reducing stroke risk and mortality). Ongoing trials have been designed to answer this. EAST-AFNET 4 (Early treatment of Atrial fibrillation for Stroke prevention Trial) has enrolled over 2,500 patients and will test whether an early, comprehensive, rhythm control using either AADs or catheter ablation prevents adverse cardiovascular outcomes compared with usual care ^93^. CABANA (Catheter Ablation versus Anti-arrhythmic Drug Therapy for Atrial Fibrillation Trial) will test the hypothesis that catheter ablation of AF is better than rate control or rhythm control with AADs at decreasing the incidence of mortality, disabling stroke, serious bleeding, or cardiac arrest ^94^.

We also do not know what the best strategy for catheter ablation of persistent AF is. For now, it is recommended that PVI alone be performed for the first ablation procedure for persistent AF. However, further trials are needed, including trials comparing PVI alone with novel ablation techniques using multi-electrode basket catheters or vests to map rotors.
